# Concrete Curing Performance Assessment Based on Gas Permeability Testing in the Lab and on Site

**DOI:** 10.3390/s22134672

**Published:** 2022-06-21

**Authors:** Lisa Ptacek, Alfred Strauss, Clémence Bos, Martin Peyerl, Roberto Torrent

**Affiliations:** 1 Department of Civil Engineering and Natural Hazards, University of Natural Resources and Life Sciences, 1180 Vienna, Austria; alfred.strauss@boku.ac.at; 2 Smart Minerals GmbH, 1030 Vienna, Austria; bos@smartminerals.at (C.B.); peyerl@smartminerals.at (M.P.); 3 Materials Advanced Services, Buenos Aires 1425, Argentina; torrent.concrete@gmail.com; 4 Materials Advanced Services, 6877 Coldrerio, Switzerland

**Keywords:** concrete curing, non-destructive testing (NDT), gas permeability testing, durability, quality control

## Abstract

Durability is an essential aspect of the lifetime performance of concrete components. The adequate surface quality and thus the service life of concrete can be achieved, among other things, by appropriate curing during hydration. To measure and control the curing quality, appropriate procedures are required. Gas permeability allows conclusions to be drawn about the porosity of concrete, which has a significant impact on durability. In this contribution, the effect of different curing methods on gas permeability is presented with the help of laboratory and on-site tests, showing that inadequate curing leads to increased permeability in the near-surface area of concrete. The measurement results of concrete samples and components with the same composition but varying curing treatment are compared and evaluated. Influences such as concrete composition and environmental factors on the quality of concrete are observed, and recommendations are made for a reliable assessment of the surface quality as a result of the investigated curing measures.

## 1. Introduction

### 1.1. Motivation

The durability of concrete has received increasing attention in recent years. In the past, it was assumed that concrete structures are maintenance-free if certain basic rules of concrete technology are followed. The experience of the past decades, however, showed that only minor deviations from these rules as well as incorrectly assessed or harsh environmental conditions can lead to considerable damage [1]. Due to the addition of ground granulated blast furnace slag and fly ash to reduce CO_2_ emissions, modern cement in particular tends toward slower reacting, which is why modern concrete requires a more extensive curing [2].

The curing of concrete is essential for the durability of a structure [3]. Various guidelines and standards as [4,5,6] give information on measures and durations for the adequate curing of concrete. However, during the construction phase, in many cases, little importance is attached to curing due to a lack of control options, tight schedules, and cost reasons. Excerpting from Prof. Neville [2]: ‘*Curing concrete is the lowest of low-tech operations… it is seen by many as a silly operation, a non-job…and bad curing does not show…. If I emphasize ensuring curing, it is because curing can make all the difference between having good concrete and having good concrete ruined by the lack of a small effort*’.

Different to bad compaction that ‘shows’, lack of curing does not show; therefore, the finding of test methods for proving sufficient curing is of great interest.

Numerous methods are already cited in the literature to evaluate the curing efficiency. In addition to standardized methods such as the determination of the abrasion resistance [7], the compressive strength [8] or the rate of absorption [9,10], alternative methods were investigated that provide information about the concrete quality as a result of curing. The procedures range from monitoring during the hydration phase to determine the appropriate time for formwork removal [11] to destructive procedures in the laboratory [12] to evaluate the curing effects afterwards. The authors of [13] investigate the effect of curing water availability on cement hydration using isothermal calorimetry. Consequential damage or damage processes occurring due to inadequate curing such as freeze–thaw attack or microcracks have been determined in studies with mercury intrusion porosimetry testing [14] or acoustic emission [15].

Various non-destructive methods are already being used to assess the durability of concrete structures, such as computer tomography, radar techniques and ultrasonic testing [16]. There is also the approach to combine non-destructive tests in order to obtain results for the evaluation of the curing performance [17].

Many of the methods mentioned are associated with great effort or represent the curing effectiveness inadequately. Therefore, the objective of this contribution is to present a non-destructive, simple, and low-cost test method for curing quality control.

### 1.2. Durability

According to [18], durability is defined as the capability of structures, products or materials to fulfill the requirements defined, which are determined after a specified period of time and usage. The durability and performance of concrete is reduced by indirect and direct damage processes during its entire service life. Direct damage processes on concrete are freeze–thaw attack, acid action and leaching processes. The indirect damage processes include chloride penetration and carbonation, which can lead to the corrosion of reinforcing steel [18]. The prevention of the mentioned damage processes and a sufficient durability is achieved amongst other things by:

- Durability-oriented design of the structure, e.g., keep away from attacking substances and water;
- Correct selection of the concrete raw materials and a concrete composition suitable for the job;
- Proper production and processing techniques, especially curing of the concrete;
- Passive protection of the concrete, e.g., by impregnation, coating [1].

### 1.3. Concrete Curing

The concrete cover needs to be as impermeable as possible to achieve a proper resistance against chemical and physical attack of the reinforcement steel, such as chloride and carbon dioxide [3]. Therefore, a high hydration rate of the concrete surface must be achieved. Cement can hydrate only when there is enough moisture available. Curing measures are carried out to ensure optimal humidity and temperature conditions in the first days of hydration and subsequently the achievement of the expected concrete quality [3]. Under a lack of proper curing, the capillary pores of the outer concrete surface dry out. This leads to a standstill of the hydration and eventually to plastic shrinkage, a reduced surface strength and higher surface permeability [18]. As a result of this, the concrete in the areas close to the surface, which are highly exposed to the weather and environmental conditions, are less durable [4,19,20].

The following methods are suitable for proper curing of the concrete surface, and they can also be combined:

- Keeping the concrete in the formwork;
- Covering the concrete surface with foils;
- Wetting the concrete surface;
- Application of an effective curing compound [18,21,22,23,24].

### 1.4. Permeability Testing

The durability of concrete depends heavily on the tightness of the concrete pore system, which, according to [1], can be demonstrated, among other things, by gas permeability. Permeable concrete favors transport processes in the concrete and therefore damage processes such as carbonation, chloride ingress and freeze–thaw deterioration [25]. It has been shown that there is a close relationship between the time it takes to reach a certain carbonation depth and the gas permeability. The authors of [26,27,28] developed an analytical model for the correlation of gas permeability with resistance to carbonation, while [29] found that the chloride input in concrete correlates with its gas permeability. Moreover, a linear relationship between freeze–thaw deterioration and gas permeability has been demonstrated [27]. The parameter gas permeability can cover the permeability of concrete for various corrosion and aging mechanisms and represents a suitable test method for characterizing the transport properties of concrete [30]. The gas permeability of a concrete surface depends on many key factors, including:

- Concrete mixture composition;
- The composition and physical properties of the cement and aggregates;
- Curing quality [31,32,33];
- Degree of hydration or age;
- Presence of microcracks;
- Presence of surface treatments;
- Moisture content at the time of the test [30,34,35].

Gas permeability is, therefore, a relevant durability indicator for the tested concrete [36].

The procedure for collecting and evaluating gas permeability data for determining the durability of the concrete surface is described in the Swiss standard SIA 262/1: 2019 [24].

**Figure 1:**
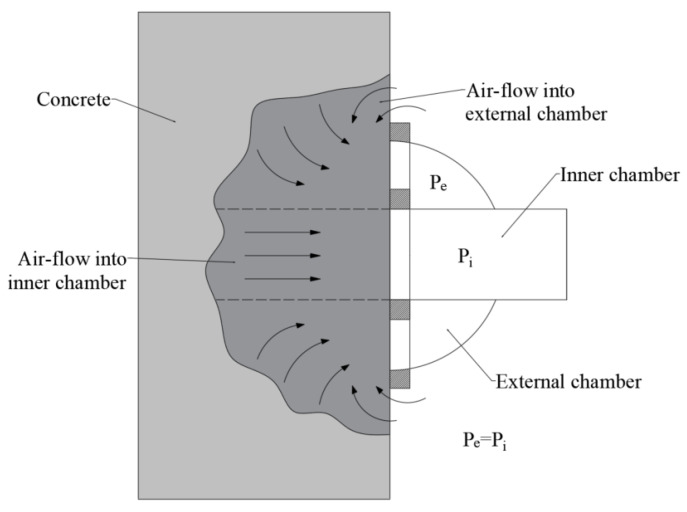
Vacuum cell, pressure regulation and air flow into its two concentric chambers [38].

As shown in Figure 1, the equipment for gas permeability testing basically consists of:

- A two-chamber vacuum cell composed of two concentric chambers (inner and external chambers);
- A control system, consisting of valves, pressure sensors and a pressure regulator that keeps the pressure of both vacuum chambers balanced during the measurement (Pe = Pi) [37].

In the inner test chamber (Ø = 50 mm) and the protective external ring surrounding this chamber, both of which are open toward the concrete surface, a vacuum is generated using a vacuum pump. Depending on the concrete and the measuring device, the vacuum is approx. 10–40 mbar. At 60 s, the inner chamber is isolated from the vacuum pump, which is the moment at which its pressure begins to build up due to the gas flowing through the concrete. The gas permeability is calculated from the change in pressure over time and other parameters. The purpose of the protective ring surrounding the test chamber (the pressure of which is permanently balanced with that of the inner chamber) is to ensure that the gas only flows into the test chamber in one dimension, which means that it only comes from inside the component (see Figure 1). For laboratory tests, cubes of at least 150 mm side length are the most suitable specimen for measuring the gas permeability coefficient kT [39]. The moisture of the concrete has a very strong influence on the gas permeability, as the water in the pores hinders the flow of air [30,33,40,41]. The concrete moisture has to be measured with an impedance-based moisture meter [42] and should be a maximum of 5.5%. This can, e.g., be achieved through drying at 50 °C for a few days prior to measurement.

In the case of in situ tests, it should be noted that according to [24], the test should be carried out when the concrete is aged between 28 and 120 days, so that the requirements for moisture content can be met. The moisture content of the concrete component to be examined must be determined in accordance with the laboratory measurements before the gas permeability measurement. In addition, it must be ensured that the air and component temperature should be at least 5 °C. Ref. [24] stipulates that 6–12 measurements need to be made per test area, which are separated by horizontal and vertical distances of at least 0.2 m. The coefficient of permeability to gas kT of hardened concrete is measured in m^2^, corresponding to the intrinsic coefficient of permeability of the material, and it is calculated with Equation (1) according to [29].

(1) $$kT={\left(\frac{V_{c}}{A}\right)}^{2}\cdot \frac{\mu }{2\cdot \varepsilon \cdot P_{a}}\cdot {\left(\frac{\ln\left(\frac{P_{a}+\Delta P}{P_{a}-\Delta P}\right)}{\sqrt{t_{f}}-\sqrt{t_{0}}}\right)}^{2},$$

where

*V_c_* = volume of the inner test chamber (m^3^);
*A* = area of the inner test chamber (m^2^);
*μ* = dynamic viscosity of air (Ns/m^2^);
*ε* = open porosity of the concrete (-) which, by default is taken as 0.15;
*P_a_* = atmospheric pressure (N/m^2^);
Δ*P* = increase in effective pressure in the inner chamber between time *t*_0_ and *t_f_* (N/m^2^);
*t*_0_ = time at which the increase in pressure is measured (s);
*t_f_* = time at which the test is finished (s).

The kT values recorded can be assessed using the permeability classes from [29]. Table 1 shows the limit values for “very good” to “very poor” concrete cover quality for the kT value measured after 28 days.

### 1.5. Objectives

With the following studies, the authors want to present and evaluate an innovative possibility to test the curing quality in a non-destructive and user-friendly way. As outlined in previous sections, the gas permeability allows making specific statements about the surface condition of concrete. Since the surface condition of concrete heavily depends on the quality of curing, the following objectives will be addressed:

1. Can curing quality be classified and evaluated using gas permeability measurements?
2. How can the curing quality on a structure be assessed? What kind of requirements must be met for reliable results with onsite measurements, considering environmental influences such as moisture, solar radiation, ambient temperature, wind, etc.?
3. Which recommendations for the implementation and analysis of gas permeability measurements for curing quality evaluation can be given on the basis of the laboratory and construction site investigations for different concrete types?

## 2. Laboratory Test Campaign

In the laboratory, it was possible to exclude influences such as wind, temperature fluctuations and solar radiation and only examine the effect of various curing conditions on concrete. By evaluating concrete samples that were treated in the laboratory under precisely defined curing conditions, connections between gas permeability and curing quality could be established.

### 2.1. Concrete and Curing Characteristics

With the involvement of a group of experts, three common types of concrete used in Austrian civil engineering were selected for the laboratory tests. Since the area of interest was particularly in road and railway construction, the types of concrete should cover as many different areas of application as possible. The names and composition of the concrete types and their fresh concrete properties according to the Austrian standard [5] are summarized in Table 2. Possible areas of application for concrete type B3 are traffic areas without de-icing agents. B5 is used for components that are exposed to spray mist containing a de-icing agent, such as bridge structures. The third type, BS1C, is used for waterproof structures directly exposed to de-icing agents.

In order to analyze various curing effects on those three concrete types, nine concrete cubes with a side length of 0.15 m were produced for each type of concrete. The cubes were subjected to three different types of curing, with a distinction being made between “good”, “no” and “bad” treatment. The applied curing conditions were discussed in advance in a panel of experts. Based on empirical values, it was assumed that with surface protection and a moderate temperature of 20 °C and a relative humidity of 60%, satisfactory concrete properties can be achieved (“good curing”). On the other extreme, being unprotected under an ambient temperature of 30 °C and a lower relative humidity of 40% should simulate more extreme conditions for “bad” curing. As can be seen in Table 3, variations for the curing types were made in the storage conditions with regard to storage with or without foil protection and also in terms of temperature and relative humidity. Since the duration of the curing treatments also influences hydration, the curing was carried out with different durations of one, three or seven days. All samples were then dried at 50 °C for three days to ensure dry samples for the measurements.

### 2.2. Measurements and Monitoring

Since there is the possibility of measurement deviations due to the application of different gas permeability devices, two devices were used for the measurements in order to compare their results, including the PermeaTORR device from Materials Advanced Services [44] and the Torrent Permeability Tester from Proceq SA [45].

Before the start of the measurements, the instruments were calibrated at least two times, until the recorded maximum pressure did not exceed 5 mbar nor did it differ by more than 0.5 mbar from the previous calibration measurement, according to [24].

A minimum of three faces per sample were measured, including the top surface that was not covered by the formwork during concreting and two lateral faces that were protected by the formwork. The measurement of two “formwork faces” was considered sufficient, since the scatter of the results between two faces is very small, and a further measurement would change the standard deviation only slightly.

As recommended in [24], the moisture content of each sample was measured with a impedance-based moisture meter CMEXII from Tramex [46] before measuring kT. To ensure the creation of a sufficient vacuum and protect the instrument, the existing loose dust on the surface was removed with a brush. In case a repetition of a measurement might be needed, due to abnormal results or to find if the permeability changes with time, the outer perimeter of the vacuum cell was marked on the surfaces. In addition to the permeability and moisture measurements, the compressive strength at the concrete age of 28 days was measured for all samples to determine whether the minimum requirements for the concrete types examined were met and to see to what extent the strength of the samples changes because of the varying curing treatments.

### 2.3. Findings

**Figure 2:**
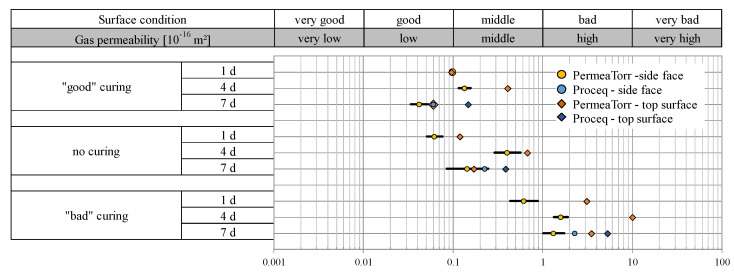
Gas permeability kT [10^−16^ m^2^] of the B3 test samples depending on the curing quality.

**Figure 3:**
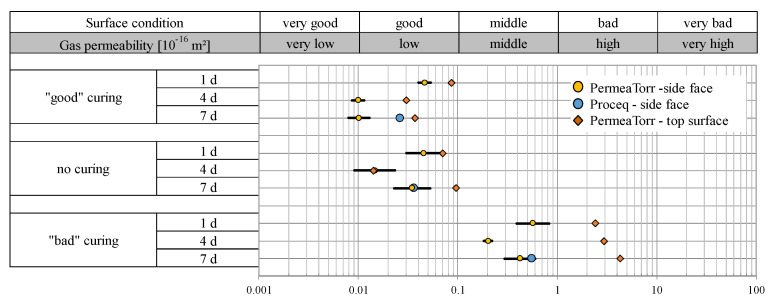
Gas permeability kT (10^−16^ m^2^) of the B5 test samples depending on the curing quality.

**Figure 4:**
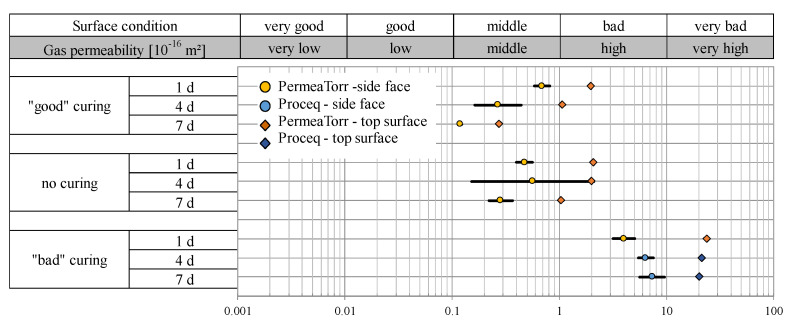
Gas permeability kT (10^−16^ m^2^) of the BS1C Plus test samples depending on the curing quality.

According to [29], the results of the permeability measurements are presented in a diagram which refers to the permeability classes. For a clear visualization of the results, the geometric mean values of the coefficient of gas permeability (kT) values in 10^−16^ m^2^ are entered as points on a logarithmic scale also showing segments representing ± the standard deviation of the logarithms of the results. A high gas permeability corresponds to a poor surface quality and vice versa. All results of a series are summarized in a separate graph for further analysis. Series B3, B5 and BS1C Plus data are shown in Figure 2, Figure 3 and Figure 4, respectively. Each graph includes the measured kT values, divided into “good”, “no” and “bad” cured samples depending on the duration of the curing treatment. When presenting the results, the focus was not only placed on the classification into surface classes but also on differentiating the results of the two devices used and the distinction of the values from measurements at the side and the top faces of the samples, which are displayed with different symbols.

#### 2.3.1. Permeability Properties of Concrete B3

Clear differences in the results of the three curing types of the samples of concrete type B3 are visualized in Figure 2. As expected, the kT values of the samples with “good” curing, shown in the first row of Figure 2, are in a good to middle surface condition class, while the values of the samples with “bad” curing, represented in the last row, are in the bad surface condition range, i.e., have a one to two orders of magnitude higher gas permeability. The samples that were not subjected to any curing treatment (middle row in Figure 2) show a middle to good surface condition, but on average, they have a slightly higher permeability than the samples with “good” curing. It can be confirmed that the duration of the curing treatment has an influence on the gas permeability. The sample with the longest treatment (7 days) of the “well”-treated samples had the lowest kT values. It follows that the protection of a sample with an adequate duration has a positive effect on the quality of the concrete. The top surfaces, marked as squares in Figure 2, tend to have a higher gas permeability, as the surface dries out faster than the side faces (round symbols) protected with the formwork and is therefore more porous. A settlement effect can also contribute to the higher permeability of the top surface as cast.

The Proceq permeability tester (blue symbols) was only used on samples with 7 days of curing. By comparing the results of the two gas permeability devices used, it can be seen that the kT values of the Proceq device are generally higher, which was a difference that was detected also by other researchers [47].

In parallel to the gas permeability measurements, the moisture content (%) of the samples was recorded according to Table 4. As listed in the third column, the average moisture decreases with increasing age of the concrete and with increasing poor curing. While the moisture content is almost the same for all samples after one day of curing, it differs more and more with increasing age in the three types of curing. Samples with “good” curing still have a moisture level of 3.6% after 7 days of curing and 3 days of drying treatment, while poorly cured samples with same curing duration only have a moisture level of 3.2%.

An influence of the curing can also be demonstrated by determining the compressive strength after 28 days. The last column of Table 4 shows the compressive strengths for all three curing types. “Good” curing samples show the highest compressive strength with 41.1 N/mm^2^, while the strength of the “bad” cured samples is the lowest with only 36.5 N/mm^2^.

#### 2.3.2. Permeability Properties of Concrete B5

As shown in Figure 3, the measured kT values of the samples of concrete type B5 (w/c = 0.48) are lower for all curing types compared to series B3 (w/c = 0.53). On average, they have a better surface quality, which suggests that the concrete composition has a significant influence on the gas permeability.

With series B5, it can be demonstrated that a “good” curing has a positive effect on the surface quality over the long term, as already could be concluded with series B3. The lowest kT values were measured in samples with “good” curing and a curing period of 4 and 7 days with about 0.01 × 10^−16^ m^2^. Samples with no curing, such as the samples with “good” curing, are in the good surface quality range with slightly higher kT values between 0.01 and 0.1 × 10^−16^ m^2^. The results of the “badly” cured samples are in the middle to bad quality range reaching from 0.2 to 5 × 10^−16^ m^2^ depending on the curing duration and the used device. Especially, the top surfaces (orange squares in Figure 3) of the samples with “bad” curing show that the quality decreases with the duration of the poor treatment.

In this series, too, it can be confirmed that the permeability tester from Proceq SA delivers higher kT values than the PermeaTORR device.

The moisture contents of the concrete type B5 are listed in Table 5 for the three curing types with different curing durations. They partly show a different effect than the samples of the B3 series. Samples with “good” or no curing show higher moisture content with increasing treatment time. “Bad” curing leads to drier samples with longer lasting curing duration. The highest humidity with 4.3% could be measured on “well” cured samples with 7 days of curing treatment followed by 3 days of drying. The lowest moisture content was found after 7 days of “bad” curing and 3 days of drying, with 3.4%.

As with series B3, “good” curing has a positive effect on the 28 days compressive strength. In Table 5, “good” curing samples from series B5 show a compressive strength of 49.7 N/mm^2^, while “badly” cured samples only achieve a strength of 38.9 N/mm^2^.

#### 2.3.3. Permeability Properties of Concrete BS1C Plus

The results of the gas permeability coefficient measurements for series BS1C Plus are presented in Figure 4. The measured values of this series also clearly confirm the influence of the curing on the kT value and the surface quality.

While the measured values of the samples with “good” and no curing are in the range of a middle to bad surface quality, reaching from 0.1 to 2 × 10^−16^ m^2^, the condition of the “bad” cured samples is correspondingly worse and is in the bad to very bad range between 2 and 12 × 10^−16^ m^2^. As it was already evident in the case of series B3 and B5, the extended “good” curing duration up to day 7 also has a positive effect on the surface quality. As with the other two series, the kT values of the top surfaces of the BS1C Plus samples are higher than those of the side faces.

The samples in this series were examined with the PermeaTorr device. Only the “bad” cured samples with a curing period of 4 and 7 days were measured with the Proceq SA permeability tester. These measurements have the highest permeability values, as shown with the blue symbols.

The average moisture in the BS1C Plus samples is given in Table 6. The values are similar to those of the B5 series, where the 7-day lasting “good” curing results in a moisture level of 4.1%. The moisture of the samples without curing treatment hardly differs due to the curing time, while the “bad” cured samples show the lowest moisture with 2.7% after 7 days of treatment.

The samples of the concrete type BS1C Plus achieve a compressive strength after 28 days of 33.7 N/mm^2^ at “good” curing conditions and 29.1 N/mm^2^ at “bad” curing conditions (Table 6). It can be concluded that also with this series, the curing type affects the compressive strength of the samples.

#### 2.3.4. Summary of the Laboratory Findings

**Figure 5:**
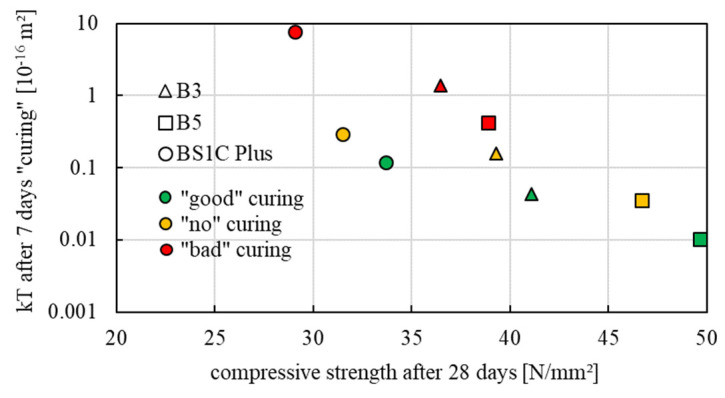
Relation between kT (10^−16^ m^2^) after 7 days “good”, “no”, “bad” curing and the compressive strength after 28 days of B3, B5 and BS1C Plus test samples.

In all three series, an effect of the curing quality on the gas permeability could be demonstrated. Samples with “good” curing generally had a lower kT value than samples with “no” or “poor” curing. As shown in Figure 5, there is a correlation between the permeability coefficient kT (geometric mean) and the compressive strength (arithmetic mean). Considering the kT values of the three concrete series as a function of the curing conditions after 7 days of curing, samples with higher gas permeability have a lower compressive strength. Therefore, better curing results in a low permeability coefficient and increases the compressive strength of concrete. On average, the BS1C Plus series achieved the highest gas permeability and therefore the worst surface condition and at the same time had the lowest compressive strength. This may be due to the higher content of additions, the hydraulic contribution of which is not very significant in the first 7 days, even under “good” curing conditions. The samples of series B5 had the highest compressive strength compared to B3 and BS1C, had lower kT values and thus achieved a better surface condition on average. The B3 series results were in the middle with regard to compressive strength and gas permeability. Overall, the laboratory tests confirm that the quality of the curing influences both the gas permeability and the compressive strength of concrete and that the type of concrete plays an important role in the evaluation of the curing quality.

## 3. On Site Test Campaign

Under controlled laboratory conditions, clear differences in the gas permeability of samples with varying curing treatments could be determined. In construction site conditions, components are exposed to a wide variety of environmental factors, such as solar radiation, temperature fluctuations, rain, wind, etc., which also strongly influence the development of the pore structure of concrete during the early hydration phase and therefore the permeability. The suitability of the gas permeability measurement for on-site evaluation of the curing quality was investigated by further tests directly on concrete structures. Concrete walls of two objects were examined at two different construction sites at different times of the year (summer and late autumn) in order to check the influence of the environmental conditions and the concrete composition on the measurement results.

### 3.1. Object 1—Measured in Summer

The first on-site investigations were carried out during dry and warm weather conditions on a concrete wall at a construction site of a railway passage of the Austrian Federal Railways in Obersiebenbrunn (48°14′43.6″ N 16°42′14.1″ E). The wall was executed with the Austrian type of concrete BS1C, which was composed according to Table 7.

Different curing areas were prepared by dividing the west-facing side of the 4 × 11 m large concrete wall into three parts, each of which was given a different curing treatment by the construction company. A distinction was made between “good” (curing A), “moderate” (curing B) and “bad” curing (curing C). The area with “bad” curing was left in the formwork for one day with no further treatment afterwards. The formwork of the “moderately” cured area was removed after four days, and the “good” area was left in formwork for seven days. Both the “good” and the “moderate” areas were encased in fleece for three days after removing the formwork and were sprinkled with water in the mornings. The outside temperature during the curing period was on average 22.5 °C, and the air humidity was 67.3%. The gas permeability tests on the concrete wall were carried out 13 days after concreting.

**Figure 6:**
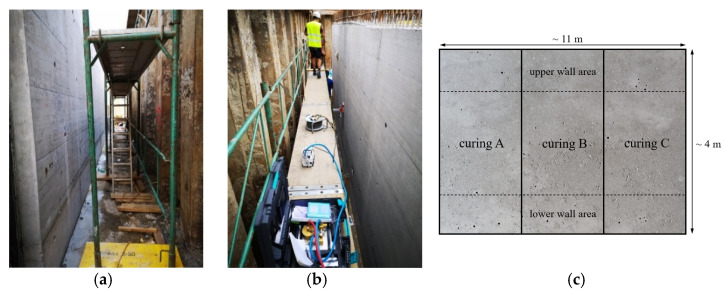
(**a**) Lower and (**b**) upper area of the concrete wall on the construction site, (**c**) division of the measured wall areas according to curing type.

As pictured in Figure 6a, the side of the wall to be examined was close to a sheet pile wall, which is why the lower area of the concrete wall was better protected from environmental influences such as solar radiation and wind than the upper area. In order to take the more humid conditions in the lower area of the wall into account, the measurements were carried out both in the upper and in the lower part and were evaluated separately. A sketch of the division of the areas can be seen in Figure 6b.

**Figure 7:**
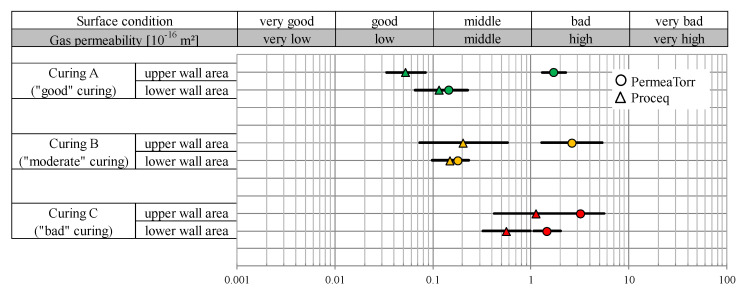
Gas permeability kT (10^−16^ m^2^) of the BS1C concrete wall depending on the curing quality.

As performed in the laboratory, the measurements were carried out with both the PermeaTorr and the Proceq devices. The results of the two devices are shown separately in Figure 7. The kT values in Figure 7 were given separately for curing A (“good” curing area), curing B (“moderate” curing area) and curing C (“bad” curing area) and for the lower and upper wall area. Each given kT value represents the geometric mean value of the results of three measurements taken per area. The graphic shows that the surface condition with curing A (“good”) is generally in the good to middle range, while curing B has a medium quality and curing C is in the middle to bad surface condition class. It is noticeable that the Proceq device measures a significantly higher gas permeability than the PermaTorr device, especially in the upper wall area. This effect could already be observed with the laboratory samples. The significantly poorer values in the upper wall area can be explained by the more intensive exposure of this area to wind and solar radiation and therefore more severe drying of the concrete surface. Since there was a more humid and shadier environment in the lower wall area, lower kT values were measured. This effect is confirmed by the moisture meter results. The humidity in the upper area averaged 3.6%, while an average of 4.1% was measured in the lower area.

The results of the construction site tests show that the quality of the curing can be measured under natural environmental influences by means of gas permeability. It can also be seen that exposure and environmental conditions have an impact on the development of concrete quality.

### 3.2. Object 2—Measured in Late Autumn

Another series of tests was carried out during cooler weather on the concrete walls of a sedimentation basin at an Asfinag (Austrian highway operator) construction site near Göttlesbrunn (48°03′55.3″ N 16°41′46.0″ E). The concrete walls were cast with Austrian concrete type B7 as described in Table 8.

**Figure 8:**
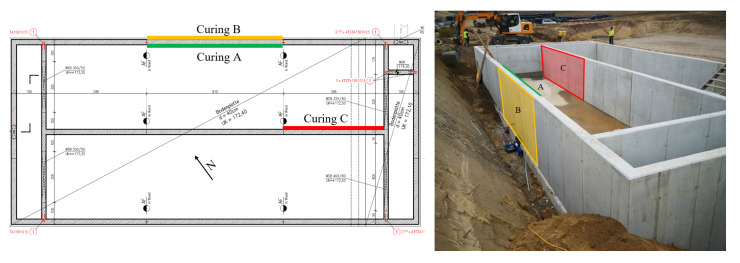
Orientation of the measured concrete walls depending on their curing treatments A, B and C.

Same as for construction site object 1, three areas with varying curing treatment were prepared. The curing for these tests was carried out on differently oriented areas. According to Figure 8, curing A (“good” curing) was carried out on the southeast side of a wall. Curing A means that the concrete was left in the formwork for 8 days. Curing B was on the opposite side, facing northeast. This area B was left in the formwork for 5 days and is referred to as “moderate” curing. Finally, a poor curing treatment (curing C) also took place on a wall facing northeast. This area was left in formwork for just three days. No further curing treatment was carried out in any of the three areas after removing the formwork.

The measurements took place when the concrete was 15 days old. The average outside temperature from concreting to the day of measurement was 6.5 °C. The humidity was relatively high at 84%.

Due to the prevailing damp weather, the moisture content of the structure was also measured. With 2.5%, the moisture content was below the maximum recommended value for gas permeability measurements. The low moisture content in the concrete was attributed to the strong wind conditions.

**Figure 9:**
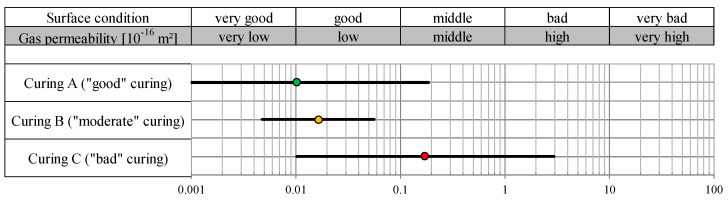
Gas permeability kT (10^−16^ m^2^) of the B7 concrete wall depending on the curing quality.

Three measurements were made per curing area with the Proceq permeability tester. The results in Figure 9 show the geometric mean kT values in 10^−16^ m^2^ with ± the standard deviations of the logarithms for each area. The tendency that the surface quality is impaired with increasingly poor curing is also confirmed in this series of tests. The values in the “good” curing area A show a very good to good surface condition, while the poorly treated wall area C only achieves middle quality. A good surface condition could be demonstrated through “moderate” curing. The results of “good” and “moderate” curing are very close to each other. It can be assumed that the favorable location of the area of curing B had a positive effect on hydration due to the adjacent moist soil, while curing A is more exposed and is therefore less protected from wind and consequently less protected from drying out. The stronger solar radiation on the southwest side in area A could be considered as a further aspect, which would also promote dehydration. However, due to the cloudy circumstances, solar radiation plays only a minor role in the period from concreting to the day of measurement. The variability of the kT data, for the three curings (especially for curings A and C) was extremely high, which was possibly due to the young age and exposure conditions prone to the development of microcracks; this experience indicates that taking at least six readings on each test area, as defined in the Swiss standard SIA 262/1: 2019 [24], is recommendable.

## 4. Discussion of the Main Findings

The laboratory tests show that the influence of the curing quality on concrete can be detected by determining the gas permeability. Concrete samples of the same composition have different properties due to different curing treatments. Sufficiently cured concrete has a lower gas permeability than untreated or insufficiently cured concrete. Furthermore, the compressive strength is reduced for laboratory samples with increasingly poor curing treatment. In addition to the curing quality, other factors, such as the composition of the concrete, are also decisive for the quality of the concrete surface. Therefore, the results of the examined types of concrete differ from each other when the curing treatment was carried out in the same way. While the concrete type BS1C Plus generally had higher kT values in the laboratory tests, the results for type B5 were significantly lower. Nevertheless, the trend of decreasing gas permeability with higher curing quality is reflected within all examined series.

It is not possible to set a general kT value as a threshold value for adequate curing. Rather, test results already collected for different types of concrete represent guide values for further measurements.

Different curing quality could also be clearly identified on the construction site. The same effect was found there as with the laboratory samples. Longer or better curing treatment had a positive effect on the concrete quality and thus lowered the gas permeability. However, the results of the construction site tests cannot be directly compared with the laboratory results, as the climatic conditions, the type of curing and the concrete composition differed.

When evaluating the quality of curing on a structure, numerous other factors are responsible for the surface quality of concrete in addition to the composition of the concrete. The gas permeability of structures on site was not only influenced by the concrete composition and curing quality (achieved in the laboratory due to variations in ambient temperature, humidity, and protection against drying out) but also by exposure to wind, solar radiation and precipitation. Concrete surfaces exposed to sunlight and wind showed a higher gas permeability than surfaces that were protected against these influences. Environmental influences can have both positive and negative effects on the hydration of the concrete. Their effect is reflected in the results of the gas permeability.

In addition, when determining the gas permeability, the age of the concrete must be taken into account, which according to the Swiss standard [24] has at least to be 28 days. The measurements of this study were already carried out approx. 14 days after concreting. A difference in the quality of the concrete due to the curing treatment was nevertheless measurable. However, the measured values should not be used as a reference for older or newer structures.

Since a single gas permeability measurement only captures a small sub-area, several measurements need to be carried out for the overall assessment of a component in order to achieve reliable results. The measurements should not be carried out before the concrete is 14 days old, as young concrete still has a very high level of moisture, which may falsify the results. In addition, the humidity of the investigated component should be determined before each permeability measurement so that the recommended limit humidity of 5.5% is not exceeded. If the limit of 5.5% is exceeded, it is advisable to repeat the measurements later once the concrete has dried sufficiently.

As already mentioned, no general recommendation for a limit gas permeability value for sufficiently cured concrete can be given. A possibility for evaluation of the results is the use of the permeability classes approach from [29] as guide values. A possible solution could be to establish reference areas where good curing conditions have been consistently applied, taking the resulting kT values as control values, not to be exceeded.

## 5. Conclusions

In the research work presented, different curing treatments were compared under otherwise almost identical conditions. It was found that the type and duration of curing has a strong influence on the gas permeability and, subsequently, on the durability of the structure. Thus, the quality of the curing of concrete structures can be measured indirectly by determining the gas permeability.

This method is characterized by the simple and non-destructive implementation of measurements both in the laboratory and on the construction site. The evaluation of the results is quick and uncomplicated. Recommendations for the representation of the permeability values and the assessment of the surface quality are already available.

In summary, the following main statements could be derived from the investigations carried out:

- Investigations in the laboratory have shown that the gas permeability measurement method is a promising and reliable method for evaluating the curing quality of concrete.
- The comparison of a “good” with “no” or a “poor” curing treatment is reflected in the permeability coefficient. In addition to the curing conditions, the gas permeability also depends on the composition of the concrete, which is why no general limit values can be specified for the curing quality evaluation.
- The higher permeability due to inadequate curing is accompanied by lower compressive strength than for optimal curing.
- The construction site tests show that the gas permeability measured directly on the structure also provides reliable information on the curing quality of concrete.
- Environmental influences play a major role in on-site measurements, since the concrete quality can be influenced both positively and negatively by factors such as wind, temperature, and humidity. For this reason, these factors must be considered when selecting the measurement points, the time of measurement and when evaluating the curing quality.
- For site testing, it is recommended to perform at least six individual measurements of kT per testing area, as set in the Swiss standard SIA 262/1:2019 [24].

As the measured permeability coefficient not only depends on curing conditions, but also on the type of concrete and other influencing factors, a direct measurement of the curing quality is not possible. One possibility would be to set a limit value in the planning phase as a minimum requirement for achieving the desired concrete quality. This limit value could be determined on the basis of reference measurements on similar concrete components with the same compositions. To implement this approach, however, further comprehensive investigations would be necessary in order to obtain a sufficient sample size for a reliable determination of these limit values.

In a follow-up research project, further investigations are currently being carried out on concrete structures with different properties and under varying climatic conditions with gas permeability measurements to evaluate the curing quality. At the same time, reference values are collected from test samples with the same formulation and optimal curing conditions for evaluating the construction site results. This promising study still requires extensive research to determine the reliability of this type of assessment.

## Figures and Tables

**Table 1:** Classification of the quality of the concrete cover according to kT [29].

Classification of the Quality of the Concrete Cover		kT Measured at 28 Days [10^−16^ m^2^]
1	very good	kT < 0.01
2	good	0.01 ≤ kT < 0.1
3	normal	0.1 ≤ kT < 1.0
4	bad	1.0 ≤ kT < 10
5	very bad	kT ≥ 10

**Table 2:** Material properties of the examined concrete types in the course of the laboratory tests.

(Austrian) Concrete Name	B3	B5	BS1C Plus
Strength class	C25/30	C30/37	C25/30
Cement (kg/m^3^):	290 (CEM II/A-M (S-L) 42.5 N)	320 (CEM II/A-M (S-L) 42.5 N)	258 (CEM I 42.5N SR0 WT27 C_3_A-free)
Additions (kg/m^3^):	40	40	103
additions type 2 according to EN 206			
Total water (kg/m^3^)	171	170	165
w/c-ratio	0.53	0.48	0.48
Aggregates (kg/m^3^):			
Gravel 16/32 round grain	457	451	333
Gravel 8/16 round grain	292	289	333
Gravel 4/8	311	307	298
Sand 0/4 round grain	770	761	792
Air-entraining agent (m%-Cement):			
Mapei Mapeair LP 100	0.30–0.97	0.00–0.50	0.00–1.20
Mapei Mapeair LP 200		0.00–0.50	0.00–0.70
Sika Addiment LPS A NEU			0.00–0.15
Superplasticizer (m%-Cement):			
Mapei Dynamon LZF	0.00–0.31	0.20–0.39	0.28–0.64
Sika VC4030Ultra	-	-	0.00–0.15
Air content (fresh concrete) (%)	2.6–3.6	2.5–4.2	5.8–8.0
Bulk density (fresh concrete) (kg/m^3^)	2379–2431	2362–2442	2224–2313
Fresh concrete temperature (°C)	21.5–22.9	21.8–24.1	19.8–23.3

**Table 3:** Curing types for the laboratory tests, see also [43].

Curing Type	Storage	Temperature	Relative Humidity
Curing conditions until day 1, 3 or 7			
good	in foil	20 °C	60%
no	without foil	20 °C	60%
bad	without foil	30 °C	40%
Curing conditions from day 1, 3 or 7			
all types	Storage at 50 °C for 3 days		

**Table 4:** Average moisture and compressive strength after 28 d tested on 15 cm cubes for series B3.

Curing Type	Curing Duration (d)	Average Moisture on Measurement Day (%)	Compressive Strength after 28 d (N/mm^2^)
good	1	4.0	41.1
	4	3.8	
	7	3.6	
no	1	4.0	39.3
	4	3.4	
	7	3.3	
bad	1	3.9	36.5
	4	3.1	
	7	3.2	

**Table 5:** Average moisture and compressive strength after 28 d tested on 15 cm cubes for series B5.

Curing Type	Curing Duration (d)	Average Moisture on Measurement Day (%)	Compressive Strength after 28 d (N/mm^2^)
good	1	3.6	49.7
	4	4.1	
	7	4.3	
no	1	3.7	46.7
	4	4.0	
	7	4.1	
bad	1	3.7	38.9
	4	3.4	
	7	3.4	

**Table 6:** Average moisture and compressive strength after 28 d tested on 15 cm cubes for series BS1C Plus.

Curing Type	Curing Duration (d)	Average Moisture on Measurement Day (%)	Compressive Strength after 28 d (N/mm^2^)
good	1	3.5	33.7
	4	3.9	
	7	4.1	
no	1	3.6	31.5
	4	3.8	
	7	3.6	
bad	1	3.2	29.1
	4	2.9	
	7	2.7	

**Table 7:** Material properties of the examined concrete type of object 1.

(Austrian) Concrete Name	BS1C
Strength class	C25/30
Cement (kg/m^3^):	275 (CEM I 42.5N SR0 WT27 C_3_A-free)
Additions (kg/m^3^): additions type 2 according to EN 206	80
Total water (kg/m^3^)	165
w/c-ratio	0.49
Aggregates (kg/m^3^):	
Gravel 16/32 round grain	442
Gravel 4/16 round grain	619
Sand 0/4 round grain	705
Air-entraining agent (kg/m^3^):	1.53
WTB Air 102	
Superplasticizer (kg/m^3^):	2.17
WTB Plast 100/3	
Air content (fresh concrete) (%)	5.8
Bulk density (fresh concrete) (kg/m^3^)	2290
Fresh concrete temperature (°C)	20

**Table 8:** Material properties of the examined concrete type of object 2.

(Austrian) Concrete Name	B7
Strength class	C25/30
Cement (kg/m^3^):	330
Additions (kg/m^3^):	80
additions type 2 according to EN 206	
Total water (kg/m^3^)	178
w/c-ratio	0.43
Aggregates (kg/m^3^):	1669
Air content (fresh concrete) (%)	6.0
Bulk density (fresh concrete) (kg/m^3^)	2251
Fresh concrete temperature (°C)	30.4
